# Supplementary material for: Large-Size Suspended Mono-Layer Graphene Film Transfer Based on the Inverted Floating Method
Source: Micromachines (Basel). 2021 May 6;12(5):525. doi: 10.3390/mi12050525 (PMC8148557; doi:10.3390/mi12050525)
Supplement: Supplementary file 1 [file micromachines-12-00525-s001.zip › micromachines-1164192-supplementary.pdf]

micromachines

MDPI

Article

Large-Size Suspended Mono-layer Graphene Film Transfer based on Inverted Floating Method

Qin Wang †, Ying Liu †, Fangsong Xu, Xiande Zheng, Guishan Wang, Yong Zhang, Jing Qiu* and Guanjun Liu*

College of Intelligence Science and Technology, National University of Defense Technology, Chang Sha, 410073, China; wangqin14@126.com (Q.W)
* Correspondence: qiujing16@sina.com (J.Q), gjliu342@nudt.edu.cn (G.L)
† These authors are equally contributed.

Methods: Transferring Graphene on a hole-through base: the PMMA was spin-coated on the CVD graphene on copper foil at 1600 rpm for 60 s. Then, the PMMA/graphene was baked at 130℃ for 1 min. Because the CVD process grew graphene layers on both sides of the copper foil, the graphene layers the other side (no PMMA) of the copper foil were removed by absorbent cotton. The copper foil was then removed by a ferric chloride solution for 1.5 h. To rinse the graphene after copper etching, the copper etchant was changed three times with fresh deionized water. After the DI water rinsing, the PMMA/graphene stack was left on the DI water for 12 h to remove any impurities and residue. Afterwards, the PMMA/graphene stack was scooped up onto a through-hole stainless steel base. Then, the PMMA was removed using the IFM method.

The schematic diagram of the transfer process is shown in Figure 1 below.

Citation: Wang, Q.; Liu, Y.; Xu, F.; Zheng, X.; Wang, G.; Zhang, Y.; Qiu, J.; Liu, G. Large-Size Suspended Mono-layer Graphene Film Transfer based on Inverted Floating Method. *Micromachines* **2021**, *12*, 525. https://doi.org/10.3390/mi12050525

Academic Editor: Ha Duong Ngo

Received: 15 March 2021
Accepted: 21 April 2021
Published: date

Publisher's Note: MDPI stays neutral with regard to jurisdictional claims in published maps and institutional affiliations.

Copyright: © 2021 by the authors. Licensee MDPI, Basel, Switzerland. This article is an open access article distributed under the terms and conditions of the Creative Commons Attribution (CC BY) license (http://creativecommons.org/licenses/by/4.0/).

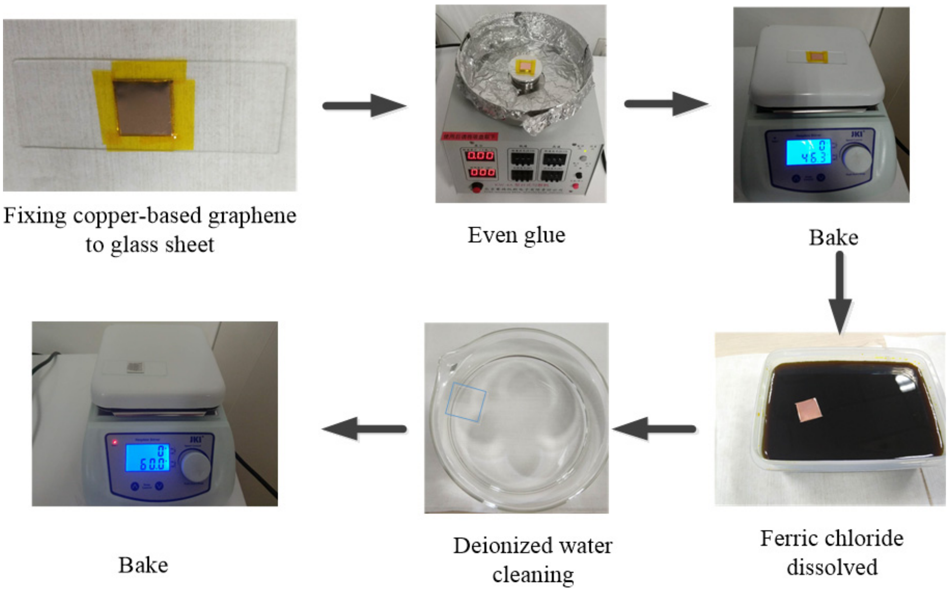

Figure S1. The schematic diagram of the transfer process.

Method 1 is the traditional dipping method. Method 2 combines the previous damage mechanism analysis to reduce the stress of the drying process and reduce the rupture of the suspended graphene film by using a liquid with low surface tension. The analysis of the damage mechanism of the graphene shows that the rupture of the graphene film mainly occurs the stage at which the three-phase interface is formed. Furthermore, in combination with a low-stress solution while avoiding the formation of a three-phase interface, IFM experiment was designed to further transfer large-size suspended graphene. Finally, the quality of the improvement method is judged by the coverage of the trans-

ferred suspended graphene film. The results of the three sets of experiments are as follows, inside the red circle is the complete suspended graphene film, and inside the blue circle is the broken.

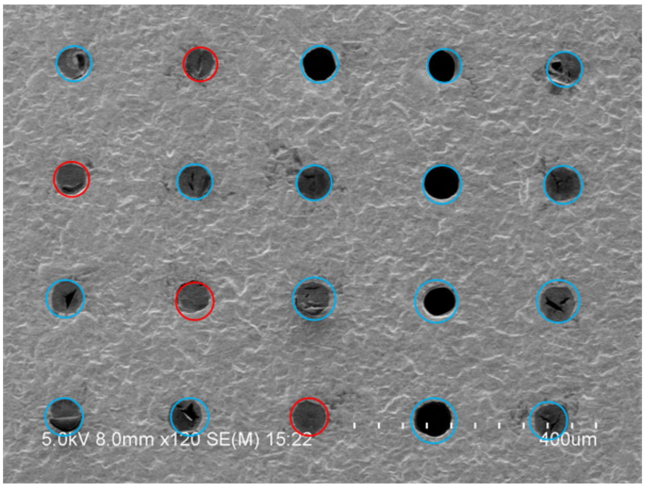

Figure S2. SEM image of 50μm suspended graphene obtained by method 1.

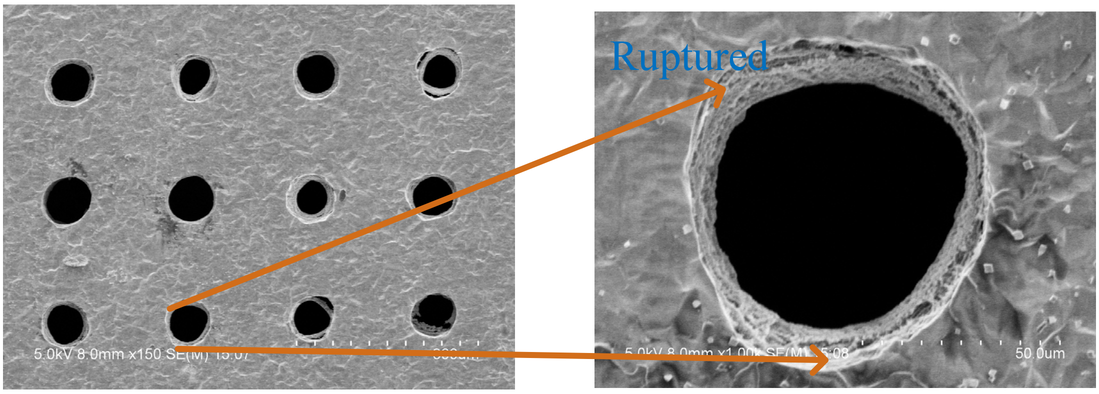

Figure S3. SEM image of 80μm suspended graphene obtained by method 1.

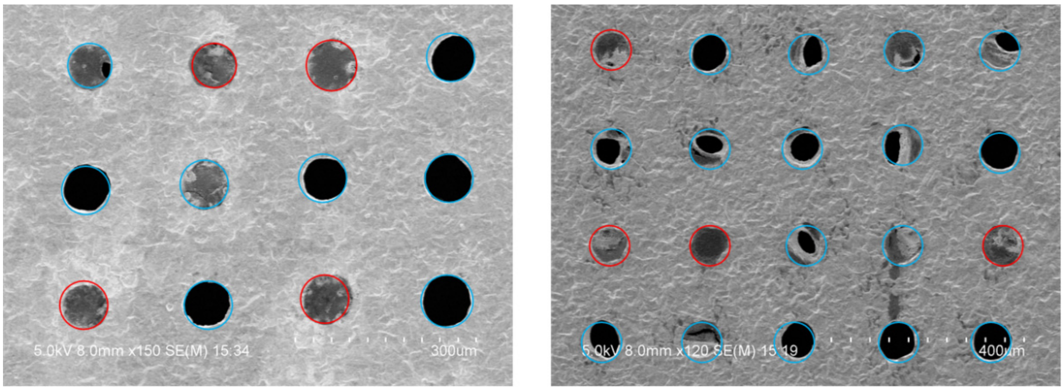

Figure S4. SEM image of 50μm and 80μm suspended graphene obtained by method 2.

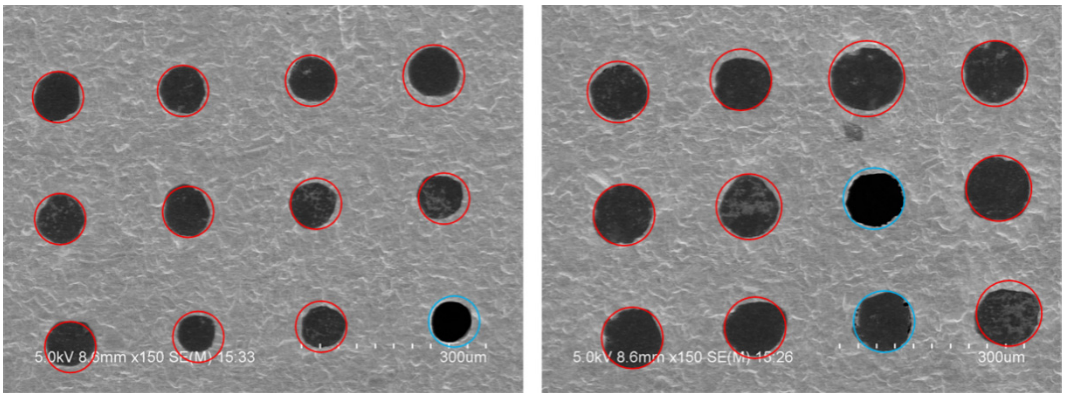

Figure S5. SEM image of 50μm and 80μm suspended graphene obtained by method 3.

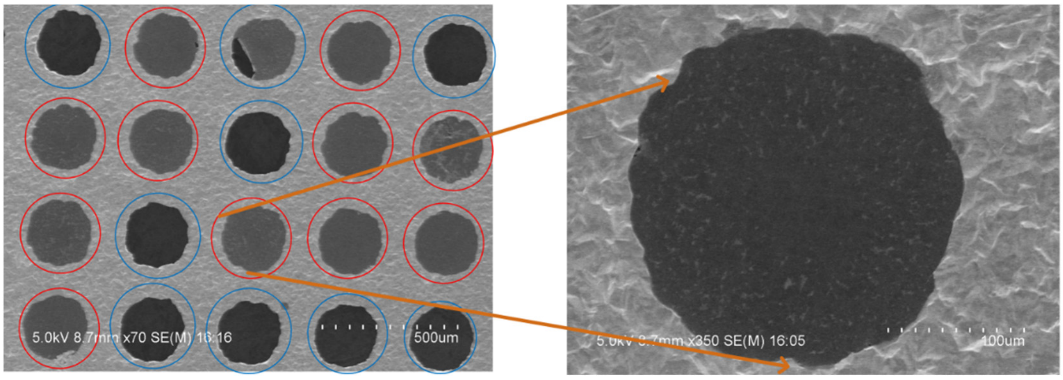

Figure S6. SEM image of 200μm suspended graphene obtained by method 3.

Table S1. Transfer success rate obtained from each method.

	50μm	80μm	100μm	200μm	500μm
Method 1	42/210 （20%）	20/210 （10%）			
Method 2	80/210 （38%）	48/210 （23%）	6/165 （4%）		
Method 3	157/210 （75%）	147/210 （70%）	104/165 （63%）	54/125 （43%）	1/35 （3%）
